# Tumor expression of miR-448 is a prognostic marker in oral squamous cell carcinoma

**DOI:** 10.1186/s12885-020-07243-z

**Published:** 2020-08-12

**Authors:** Hui Wei, Kang Yu, Yongheng Liu, Lili Li, Guowen Wang

**Affiliations:** 1 Tianjin Medical University Cancer Institute and Hospital, National Clinical Research Center for Cancer, Key Laboratory of Cancer Prevention and Therapy, Tianjin, Tianjin’s Clinical Research Center for Cancer, Tianjin, 300060 People’s Republic of China; 2 State Key Laboratory of Medicinal Chemical Biology (Nankai University), Tianjin, 300353 People’s Republic of China

**Keywords:** MiR-448, Oral squamous cell carcinoma, Prognosis, Overall survival, Disease-free survival

## Abstract

**Background:**

Prognosis is poor for patients with malignant progression such as distant metastasis of oral squamous cell carcinoma (OSCC). Evidence indicates that miR-448 promotes the proliferation and inhibits apoptosis of OSCC cells. Therefore, we aimed to investigate the function of miR-448 to predict tumor progression and prognosis of OSCC.

**Methods:**

Real-time quantitative reverse transcription PCR was used to measure miR-448 expression in 221 pairs of OSCC tissues and the corresponding noncancerous tissues. Patients were diagnosed with OSCC from 2009 through 2011 at the Tianjin Medical University Cancer Institute and Hospital. Chi-squared tests were performed to assess the associations between miR-448 expression and clinicopathological parameters. Kaplan–Meier analysis was employed to evaluate the association of overall survival (OS) and disease-free survival (DFS) with miR-448 levels. Univariate and multivariate analyses were performed using the Cox proportional hazards regression model.

**Results:**

We show here that miR-448 expression was significantly up-regulated in OSCC tissues compared with noncancerous tissues (*P <* 0.01). High miR-448 expression was significantly associated with advanced T stage (*P* = 0.001), lymph node metastasis (*P* = 0.007) and higher TNM stage (*P* = 0.009). Moreover, Kaplan–Meier and univariate analyses revealed that patients with high expression of miR-448 experienced significantly shorter OS and DFS. Furthermore, multivariate analysis demonstrated that miR-448 expression was an independent prognostic factor for OS (*P* = 0.004) and DFS (*P* = 0.002).

**Conclusions:**

Our present data suggests that miR-448 may play an important role in tumor progression and serves as a prognostic marker for OSCC. Further studies are required to assess the potential value of miR-448 to contribute to personalized treatment of OSCC.

## Background

Oral cancer is a subset of head and neck cancers, which originates in the oral cavity [1]. At least 90% of oral cancers originate from squamous cells and are accordingly designated as oral squamous cell carcinoma (OSCC) [2]. Despite continuous advances in treatment strategies and diagnostic methods, the incidence and mortality rates associated with OSCC are increasing [3, 4]. The main strategy for managing OSCC is comprehensive treatment employing surgery and it has reported that the 5-year survival rate of OSCC has increased for decades [5, 6]. However, prognosis is worse for patients with OSCC with malignant progression such as distant metastasis [7]. Therefore, it is necessary to find effective biomarkers to predict tumor progression and prognosis to provide personalized treatment.

MicroRNAs (miRNAs) are endogenous noncoding RNAs (approximately 22 base pairs) that regulate the expression of mRNAs by binding to their 3`-untranslated regions [8, 9]. Through these interactions, miRNAs mediate the regulation of cellular differentiation, development, and metabolism [10]. Increasing evidence demonstrates that aberrant regulation of miRNAs plays important roles in various cancers [11, 12]. Furthermore, miRNAs may possess oncogenic or tumor suppressor activity according to cellular phenotypes and their target genes. For example, miR-187 functions as a tumor promoter in oral carcinoma by targeting BARX2 [13]. In contrast, miR-429 functions as a suppressor of OSCC by targeting ZEB1 [14].

Aberrant expression of miR-448 is frequent in several cancers, including OSCC. Recently, Shen et al. reported that miR-448 promoted the proliferation and inhibited apoptosis of OSCC cells through targeting MPPED2, which suggests that the former may contribute to the progression of OSCC [15]. However, the clinical significance of miR-448 in OSCC has not been studied. Therefore, the purpose of the present study was to investigate the value of miR-448 as a predictor of prognosis of OSCC.

## Methods

### Patients

Inclusion criteria: We included patients with complete clinicopathological data who were diagnosed with OSCC from 2009 through 2011 at Tianjin Medical University Cancer Institute and Hospital. These patients had no other malignant tumors within 5 years before treatment. Distant metastasis was not detected before treatment. Exclusion criteria: We excluded patients who died because of diseases unrelated to OSCC within 5 years after treatment or those with incomplete follow-up data because of failure to return to the hospital or a change in their contact information. Finally, 221 patients were collected excluding 3 patients who died because of cerebrovascular and cardiac disease and 6 patients who changed their contact information. Follow-up ranged from 3 months to 72 months.

Patients’ samples were collected from the tumor tissue specimen bank of Tianjin Medical University Cancer Institute and Hospital. Patients’ information was obtained from medical records. Patients’ tumor and corresponding noncancerous tissues were acquired using a standardized procedure. For patients with local relapse, recurrent specimens were used. Two pathologists independently evaluated slides according to the guidelines of the AJCC manual. All surgical procedures and other treatments were performed according to NCCN guidelines. The tissues were immediately frozen in liquid nitrogen and stored at –80°C. Patients’ medical records included gender, age, smoking status, tumor grade, tumor site, T stage, lymph node metastasis, and TNM stage. Subsequent to pathological grading, 164 cases were classified as moderately or well differentiated, and 57 cases were classified as poorly differentiated. Patients’ detailed clinical information is listed in Table 2.

The Ethics Committee of Tianjin Medical University Cancer Institute and Hospital approved this study. Written informed consent was obtained from each patient before their inclusion in the study.

### RNA extraction and quantitative RT-PCR (qPCR)

Total RNAs from cancerous and normal tissues were isolated using TRIzol reagent, and cDNA was synthesized using a universal cDNA synthesis kit. RNA levels were detected using a SYBR real-time qPCR kit. PCR reaction conditions were as follows: 42 °C for 15 min; 85 °C for 5 s; and a hold at 4 °C. The cDNA products were diluted 1:100, and 1 μl of the diluted cDNA products was used for the qRT-PCR reaction. Primer sequences are shown in Table 1. The qRT-PCR reactions were repeated three times. The relative expression level of miR-448 was normalized to that of U6 and was calculated using the 2^–ΔΔCT^ method [16]. The miR-448 qRT-PCR data were considered a continuous variable.

**Table 1:** Sequence of the primers used in this study

Primer	Primer sequences
miR-448-F	5'-TTATTGCGATGTGTTCCTTATG-3'
miR-448-R	5'-ATGCATCCACGGGCATATACACT-3'
U6-F	5'-CGCTTCGGCAGCACATATAC-3'
U6-R	5'-ACGAATTTGCGTGTCATCCT-3'

### Statistical analysis

The data were analyzed using SPSS 19.0 software. According to the median value (4.46) of the miR-448 expression level, patients were divided into high ( >4.46) and low (≤4.46) groups. Chi-squared tests were performed to assess the significance of the associations between miR-448 expression and clinicopathological parameters including gender, age, smoking status, tumor site, tumor grade, T stage, lymph node metastasis, and TNM stage. Disease-free survival (DFS) and overall survival (OS) were defined as the time from initial surgery to clinically or radiologically confirmed recurrence/metastasis or death, respectively. Kaplan–Meier analysis was employed to assess the associations of OS and DFS of patients with OSCC with miR-448 levels, and the significance of the differences between groups was assessed using the log-rank test. Univariate and multivariate analyses of the associations of clinicopathological parameters and miR-448 levels with OS and DFS were calculated using the Cox proportional hazards regression model. Hazard ratios and corresponding 95% confidence intervals were estimated. The proportional hazard assumption based on Schoenfeld residuals was tested using STATA 15.0 software. All tests were two-sided, and *P* <0.05 indicates a significant difference.

## Results

### MiR-448 is overexpressed in OSCC

**Fig. 1:**
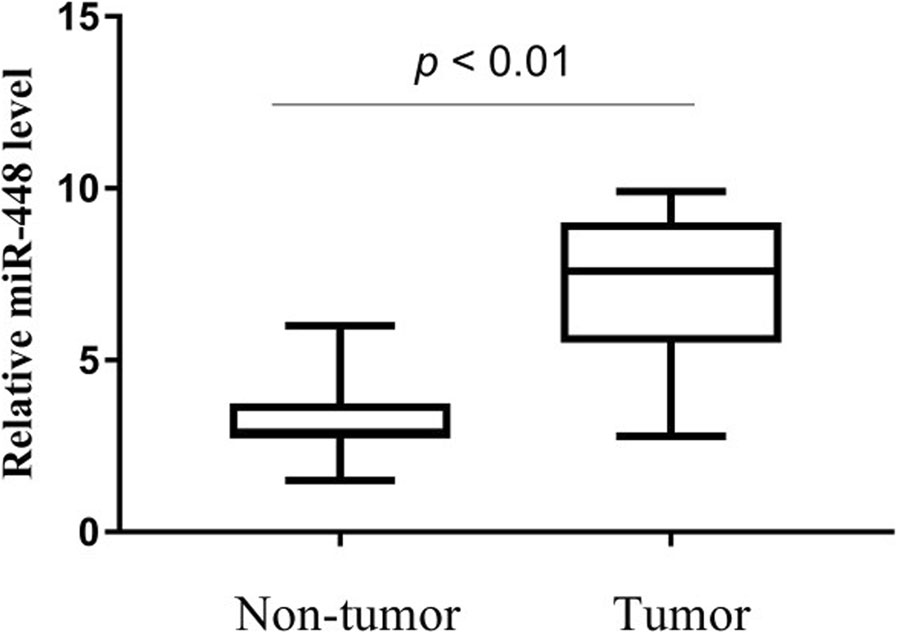
Relative miR-448 expression levels in OSCC tissues and the adjacent non- cancerous tissues from the same patient were detected by real time qPCR analysis

We used qRT-PCR to investigate miR-448 expression in 221 pairs of OSCC tissues and the corresponding noncancerous tissues. As shown in Fig. 1, miR-448 levels were significantly higher in OSCC tissues than in adjacent non-neoplastic tissues (*P<*0.01). These results suggest that miR-448 may play an oncogenic role in OSCC.

### Associations between miR-448 levels and clinicopathological features of OSCC

To investigate the oncogenic role of miR-448 in OSCC progression, we analyzed the associations between tissue miR-448 levels and clinicopathological features. The 221 patients with OSCC were divided into two groups according to the median value (4.46) of miR-448 levels. As shown in Table 2, high levels of miR-448 were significantly associated with advanced T stage (*P*=0.001), lymph node metastasis (*P*=0.007), and higher TNM stage (*P*=0.009). However, the levels of miR-448 were not associated with other clinical features such as age, gender, smoking status, and tumor site (*P*> 0.05).

**Table 2:** Associations between miR-448 and clinicopathologic variables of OSCC

Clinicopathologic variable	No.	miR-448 expression		*P* value
		Low	High	
Gender				0.412
Female	86	41	45	
Male	135	72	63	
Age (years)				0.561
<60	102	50	52	
≥60	119	63	56	
Smoking status				0.876
Non-smoker	83	43	40	
Smoker	138	70	68	
Tumor site				0.268
Tongue	62	28	34	
Non-tongue	159	85	74	
Grade				0.114
Well/moderate	164	89	75	
Poor	57	24	33	
T stage				0.001
T1–T2	145	87	58	
T3–T4	76	26	50	
Lymph node metastasis				0.007
No	154	88	66	
Yes	67	25	42	
TNM stage				0.009
I–II	161	91	70	
III–IV	60	22	38	

### Elevated levels of miR-448 are associated with poor prognosis of patients with OSCC

**Fig. 2:**
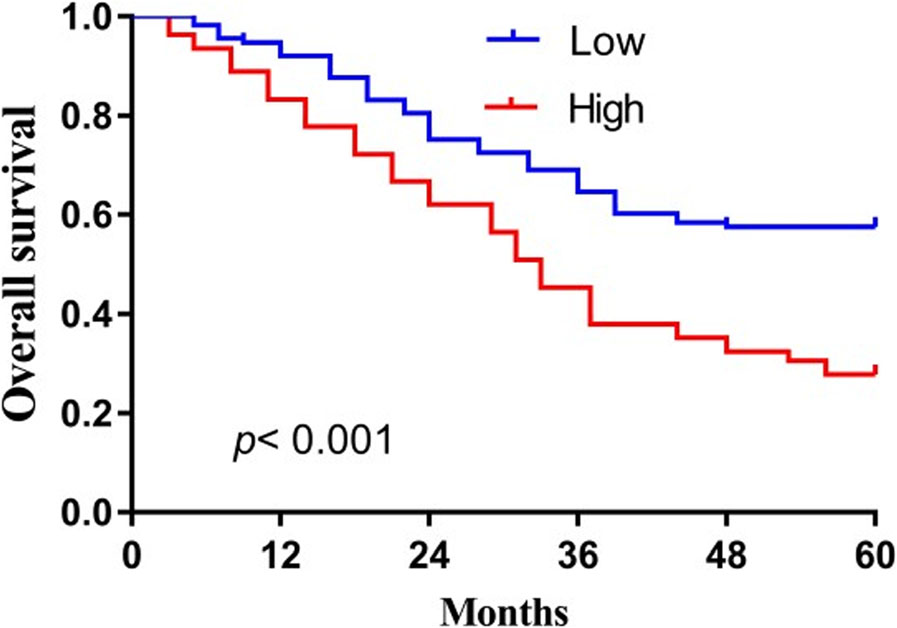
The 5-year overall survival rate of OSCC patients with high miR-448 levels was significantly poorer compared to that of patients with low miR-448 levels

**Fig. 3:**
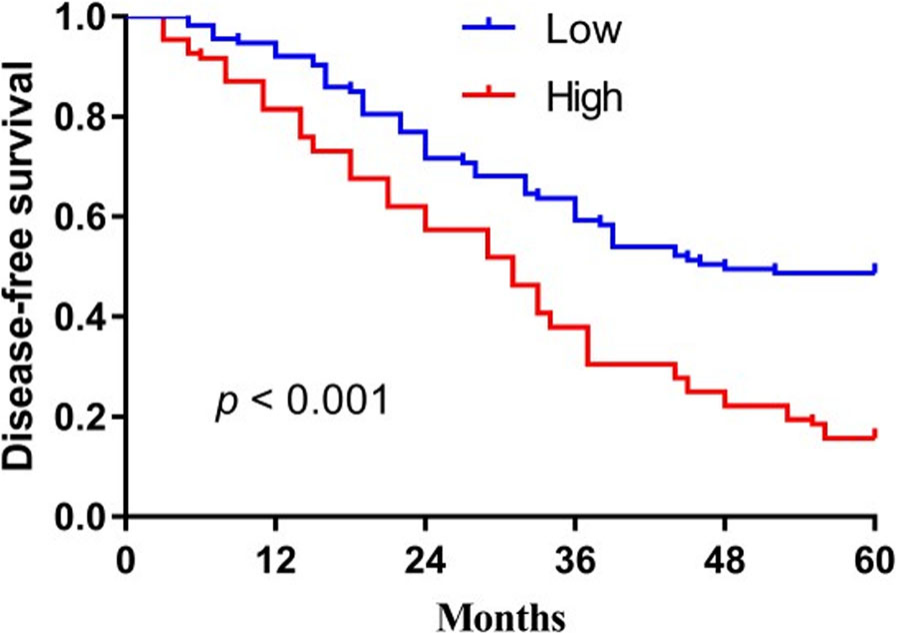
The 5-year disease-free survival rate of OSCC patients with high miR-448 levels was significantly poorer compared to that of patients with low miR-448 levels

To evaluate the prognostic value of miR-448 expression in OSCC, survival curves were generated using the Kaplan–Meier method and compared using the log-rank test. According to the median level of expression, 221 patients were divided into two groups with low or high levels of miR-448. As shown in Fig. 2, patients with OSCC with lower levels of miR-448 experienced significantly longer OS (median 68.4 months) compared with those with high levels of miR-448 (median 31.1 months, *P*<0.001). Similarly, the median DFS of patients with low or high levels of miR-448 were 46.2 months and 29.2 months, respectively (*P*<0.001) (Fig. 3).

Univariate and multivariate analyses were calculated using the Cox proportional hazards regression model and the proportional hazard assumption based on Schoenfeld residuals was tenable (*P*>0.05). Univariate analysis revealed that T stage, lymph node metastasis, TNM stage, and miR-448 expression were significantly associated with OS and DFS (Table 3). Furthermore, multivariate analysis revealed that miR-448 levels served as an independent biomarker for predicting OS (*P*=0.004) and DFS (*P*=0.002, Table 3).

**Table 3:** Univariate and multivariate analyses of overall survival and disease-free survival of OSCC patients by Cox regression analysis

Variables	Univariate analysis			Multivariate analysis		
	HR	CI (95%)	*P* value	HR	CI (95%)	*P* value
OS						
MiR-448 expression (Low/High)	3.422	1.944-5.782	0.002	4.456	2.215-6.892	0.004
Gender (Female/Male)	1.352	0.563-1.723	NS	-	-	-
Age (<60 / ≥60 years)	1.532	0.744-2.631	NS	-	-	-
Smoking status (No/Yes)	1.318	0.633-1.428	NS	-	-	-
Tumor site (Tongue/Non-tongue)	0.932	0.782-1.345	NS	-	-	-
Grade (Well-moderate/Poor)	1.784	0.742-2.342	NS	-	-	-
T stage (T1-T2/T3-T4)	2.933	1.428-5.236	0.011	2.671	1.763-6.213	0.016
Lymph node metastasis (No/Yes)	3.782	1.922-7.678	0.004	4.523	2.456-8.892	0.007
TNM stage (I–II/III–IV)	3.213	1.457-5.231	0.008	4.231	1.774-6.732	0.012
DFS						
MiR-448 expression (Low/High)	3.622	1.782-6.773	0.001	4.923	2.672-8.723	0.002
Gender (Female/Male)	1.523	0.731-1.953	NS	-	-	-
Age (<60 / ≥60 years)	1.786	0.892-2.993	NS	-	-	-
Smoking status (No/Yes)	1.554	0.458-1.889	NS	-	-	-
Tumor site (Tongue/Non-tongue)	0.782	0.336-1.231	NS	-	-	-
Grade (Well-moderate/Poor)	1.993	0.823-2.562	NS	-	-	-
T stage (T1-T2/T3-T4)	2.532	1.632-5.773	0.008	2.892	1.933-6.663	0.011
Lymph node metastasis (No/Yes)	3.922	2.213-8.442	0.003	4.872	2.773-9.032	0.005
TNM stage (I–II/III–IV)	3.552	1.642-5.833	0.006	4.521	1.883-6.521	0.009

*HR* hazard ratio, *CI* confidence intervals, *NS* non-signifiant, “-”: No analysis

## Discussion

Vigorous efforts over the past several decades have focused on identifying biomarkers that help predict prognosis of OSCC as well as to develop new therapeutic approaches [17]. However, the relatively low specificity of such markers limits their clinical application [18, 19]. Recently, the critical role of miRNAs in tumor progression gained the attention of investigators who suggest that miRNAs may serve as novel prognostic indicators of patients with cancer. Moreover, the prognostic potential of miRNAs is established for several types of cancer, including OSCC. For example, Liao et al. reported that patients with OSCC with higher levels of miR-1246 survive at much lower rates than those with lower levels [20]. They further found that high levels of miR-1246 serve as an independent predictor of poor prognosis [20]. Peng et al. reported that low levels of miR-218, miR-125b, and let-7g are associated with poor survival of patients with OSCC [21]. However, the effects of other miRNAs on OSCC are largely unknown.

The roles of miR-448 in other types of cancer are known. For example, Wu et al. reported that ectopic expression of miR-448 suppresses the proliferation, colony formation, and invasion of gastric cancer cells through the regulation of ADAM10 [22]. Shan et al. found that miR-448 exerts a tumor suppressor function through targeting DCLK1, leading to the inhibition of lung cancer cell growth and metastasis [23]. They further found that low levels of miR-448 serve as a poor prognostic factor for patients with lung squamous cell carcinoma [23]. Zhu et al. reported that miR-448 inhibits tumorigenic processes such as growth, viability, migration, and invasion of hepatocellular carcinoma cell lines through targeting ROCK2 [24]. Lv et al. found that ectopic expression of miR-448 represses the proliferation, migration, and invasion of ovarian cancer cells through targeting CXCL12 [25]. These results show that miR-448 acts as a tumor suppressor in the above tumors. In contrast, Shen et al. found that the levels of miR-448 are significantly increased in human OSCC tissues and cell lines vs controls [15]. Furthermore, miR-448 functions as a tumor promoter in OSCC through targeting MPPED2 [15]. These results reveal that the function of miR-448 varies in different types of tumors.

Our present findings are consistent with those of Shen et al. [15] in that we found that the expression of miR-448 was significantly upregulated in OSCC tissues compared with matched normal tissues. Furthermore, high levels of miR-448 were significantly associated with advanced T stage, lymph node metastasis, and higher TNM stage; and patients with higher levels of miR-448 experienced shorter OS and DFS. Moreover, high levels of miR-448 served as an independent predictor of poor prognosis of patients with OSCC.

Here we show for the first time that miR-448 could be an independent prognostic biomarker of OSCC. Our findings of the clinical significance of miR-448 expression will provide possibilities for the application of miR-448 in personalized treatment of OSCC. Our study is limited, however, by the insufficient number of informative clinicopathological parameters (e.g. lack of ECOG/WHO performance status and p16 expression).

## Conclusions

Our present data support the conclusion that miR-448 may play an important role in tumor progression and serves as a prognostic biomarker for OSCC. Further studies are required to assess the potential value of miR-448 in developing personalized treatment.

## Data Availability

All data generated or analysed during this study are included in this published article.

## Abbreviations

OSCC: Oral squamous cell carcinoma
qRT-PCR: Quantitative reverse transcription PCR
OS: Overall survival
DFS: Disease-free survival
UTR: Untranslated region
SPSS: Statistical Package for the Social Science
STATA: Software for Statistics and Data Science
HR: Hazard ratio
CI: Confidence intervals
NS: Non-signifiant
